# Silicon-Mitigated Effect on Zinc-Induced Stress Conditions: Epigenetic, Morphological, and Physiological Screening of Barley Plants

**DOI:** 10.3390/ijms26010104

**Published:** 2024-12-26

**Authors:** Marzena Mazurek, Renata Tobiasz-Salach, Barbara Stadnik, Dagmara Migut

**Affiliations:** 1 Department of Physiology and Plant Biotechnology, University of Rzeszow, Ćwiklińskiej 2, 35-601 Rzeszow, Poland; 2 Department of Crop Production, University of Rzeszow, Zelwerowicza 4, 35-601 Rzeszow, Poland; rtobiasz@ur.edu.pl (R.T.-S.); barbarast@dokt.ur.edu.pl (B.S.); dmigut@ur.edu.pl (D.M.); 3 Doctoral School, University of Rzeszow, Rejtana 16C, 35-959 Rzeszow, Poland

**Keywords:** abiotic stress, chlorophyll fluorescence, DNA methylation, heavy metals, *Hordeum vulgare* L.

## Abstract

Plants are increasingly exposed to stress-induced factors, including heavy metals. Zinc, although it is a microelement, at high concentrations can be phytotoxic to plants by limiting their growth and development. The presented research confirmed the inhibition effect of Zn on morphological and physiological parameters in barley plants. However, the effect was Zn dose dependent (50 µM, 100 µM, and 200 µM), as well as part of the plants (above ground or roots). To mitigate the negative effects of Zn, plants were sprayed with 0.1% silicon. Silicon was proven to have a positive effect on mitigating the inhibitory effects of Zn-induced stress. In most cases, an increase in both morphological (length, elongation, fresh and dry weights, and weather content) and physiological (relative chlorophyll content and fluorescence) parameters was observed. This occurrence was dependent on the Zn dose. Epigenetic analyses confirmed differences in the DNA methylation level, both between plants subjected to stress at different strengths (50 µM, 100 µM, and 200 µM Zn) and between plants sprayed with Si or not. The differences indicate that silicon affects the epigenome of barley plants, thereby modifying the response of plants to stress factors. This modification may be the basis for plants to acquire resistance as “epigenetic memory”.

## 1. Introduction

Environmental pollution and climate change mean that all organisms, including plants, in their natural environment are increasingly and more often exposed to various types of stress (biotic and abiotic). Abiotic stresses are defined as the adverse impact of inorganic factors on living organisms in a specific environment. High or low temperature, salinity, drought or flood, nutrient deficiency, or metal toxicity are a few of the main abiotic stress conditions a plant faces daily during its life cycle [1,2]. Depending on the duration and concentration, these factors contribute to the inhibition of plant growth and development, which, in turn, affects the quantity and quality of crops [3]. In the agricultural sector, abiotic stress is one of the main reasons for massive monetary and production losses [2].

Heavy metals are a major problem for plants, disrupting plant metabolism due to the continuous interaction that occurs at the cellular level [1,4,5]. Metals that have a negative impact on nature include lead (Pb), mercury (Hg), and cadmium (Cd), arsenic (As), but also metals such as copper (Cu), aluminum (Al), manganese (Mn), and zinc (Zn) [6]. Zn, although an essential micronutrient required for plant growth and development, at high concentrations can be toxic to plants [1,7,8]. Zn is important for plant growth and development, playing a crucial role in various enzymes and plant proteins in photosynthesis, hormone regulation, and nutrient uptake [1,9]. However, the accumulation of Zn in soil can result in significant environmental challenges, including a reduction in soil fertility and potential toxicity to both plants and microorganisms [5,10]. Zn accumulation in the soil can lead to significant environmental changes. High levels of Zn disrupt nutrient cycling and alter soil microbial communities, affecting ecosystem sustainability. This contamination can have significant impacts that ultimately affect the overall balance and sustainability of ecosystems [11]. Zn pollution can arise from various sources, such as agriculture, industry, and waste disposal. The primary source of Zn contamination in the soil stems from the use of Zn-containing fertilizers in agricultural production [5]. Phytotoxic levels of Zn in the soil can cause various changes in plants, such as reduced growth, photosynthesis, and respiration rates; unbalanced mineral nutrition; and increased production of reactive oxygen species (ROS) [12]. Plants exposed to phytotoxic Zn, characterized by the curling and twisting of young leaves, death of leaf tips, and chlorosis, were observed [8,13,14,15,16]. However, the decrease in germination strength [12] as well as shoot and root biomasses [7] are the most visible physiological responses of plants to Zn toxicity. The root system is the part of the plant that first comes into contact with heavy metals; therefore, it is the most exposed to the toxic effects of the components of the soil [8]. Scientific studies show that high concentrations of metals in the soil, including Zn, lead to anatomical and morphological changes in the roots [17,18]. Zn redundancy inhibits the elongation of primary roots [19] and stimulates the growth of lateral roots in the vertical direction. A reduction in root growth slows the absorption of water and minerals and, in turn, the growth of shoots. Several structural and functional abnormalities that occur in plants under the influence of phytotoxic doses of Zn ultimately lead to a reduction in their yield. This generates problems for potential growers and farmers [12].

Therefore, scientists’ research is focused on finding new methods to limit or mitigate the effects of stress in plants, including stress caused by environmental pollution with metals [20]. One of these methods is based on the application of silicon to plants exposed to stress [21,22]. Numerous scientific studies show that silicon leads to improved plant growth and plant resistance mechanisms to abiotic [22,23], and biotic stresses. Under metal-inducing stress conditions, silicon affects the translocation and distribution of metals in different parts of plants, thus allowing them to survive [24,25,26,27,28]. The potential role of silicon in alleviating stress occurs through the regulation of physiological, biochemical, and molecular processes [29]. Silicon can modify the expression of genes involved in plant physiological processes related to stress alleviation [30].

Plants respond to stress by adapting to or becoming accustomed to it, leading to long-term changes [31]. The basis of this phenomenon is the fact that under stressful conditions, specific transcription factors, signaling pathways, as well as effector proteins are activated to overcome stress [32]. More and more scientific studies [21,22,33,34] show that changes in gene expression that occur under the influence of stress conditions are the result of epigenetic changes. Epigenetic changes are defined as changes in gene expression caused by a mechanism other than a change in the basic DNA sequence [35,36]. Therefore, DNA modification of the “epigenetic type” in genomic DNA refers to a situation in which the information contained in DNA is modified without changing the nucleotide sequence [37]. Epigenetic changes include DNA methylation or histone modification affected by genetic information in turn. DNA methylation consists mostly in adding a methyl group at the fifth carbon position of a cytosine ring, and, unlike what happens in animals, plants have three sites that can often suffer methylation: CG, CHG (where H is A, C, or T), and CHH [37,38]. The epigenetic effect of changes in the DNA methylation pattern includes, among others, the mechanism of gene silencing or activation. According to the latest scientific reports [21,34], epigenetic changes in plants enable their adaptation to new environmental conditions.

Nowadays, scientific research of plants and their response to stress conditions focuses not only on morphological, physiological, or biochemical analyses but also on epigenetic ones. This allows for a comprehensive understanding of the plants’ reaction mechanisms and the acquisition of resistance to adverse stress factors. According to the above, the aim of the presented research was to indicate the impact of silicon on barley plants treated with Zn at different concentrations. The impact was determined through the morphological characteristics and biochemical and epigenetic levels. In addition, an important element of the research conducted is the use of silicon as the foliar spray form. This form of spraying allows for the quick delivery of ingredients to the plant, in the case where their uptake from the soil solution may be difficult due to the high concentration of stress-inducing ingredients.

## 2. Results

### 2.1. The Effects of Zn and Si Application on the Biomass of Barley Plants

In the research carried out, foliar silicon spraying had a mitigated effect on barley plants growing under stress conditions caused by Zn ions in different concentrations. The effects of silicon application were visible on the morphological, physiological, and epigenetic levels.

Barley plants grown under Zn-induced stress conditions are mainly characterized by a decrease in all analyzed parameters, with some exceptions. This was visible especially in the case of the above-ground parts of plants and less in their roots. Nonetheless, the stimulation effect of Zn ions was also observed in the roots. However, the effect (inhibition or even stimulation) was Zn dose dependent.

**Figure 1:**
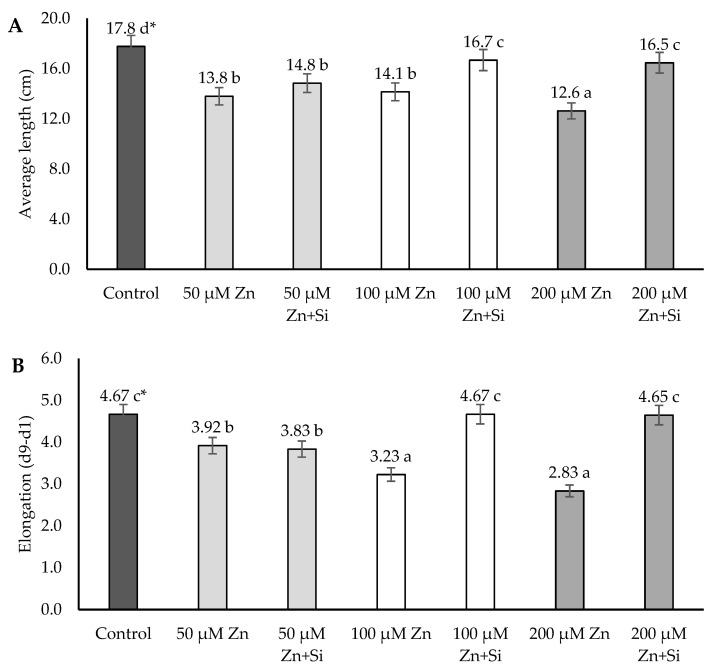
Effects of the application of Zn and Si on the length (**A**) and growth (**B**) of the above-ground barley; data are expressed as mean ± SD values. * Different letters indicate significant differences between the variants of the experiment (*p* ≤ 0.05).

The average length and elongation of the above-ground barley plants decreased with all doses of Zn, whereas the application of Zn at a high concentration (200 μM) had the highest inhibitory effect on the plant length as well as elongation (Figure 1). The average length and elongation of the plants treated with 200 μM Zn were almost one-third reduced than the control plants (Figure 1), while Zn at concentrations of 50 μM and 100 μM led to a reduction in the length and elongation parameters compared to control plants, more or less, but not so drastically.

The application of silicon mitigated the negative effect of Zn ions. Si led to the increase in all growth parameters (average of the length as well as elongation). It was statistically confirmed in the case of high dose of Zn (200 μM) as well as moderate dose (100 μM Zn).

**Figure 2:**
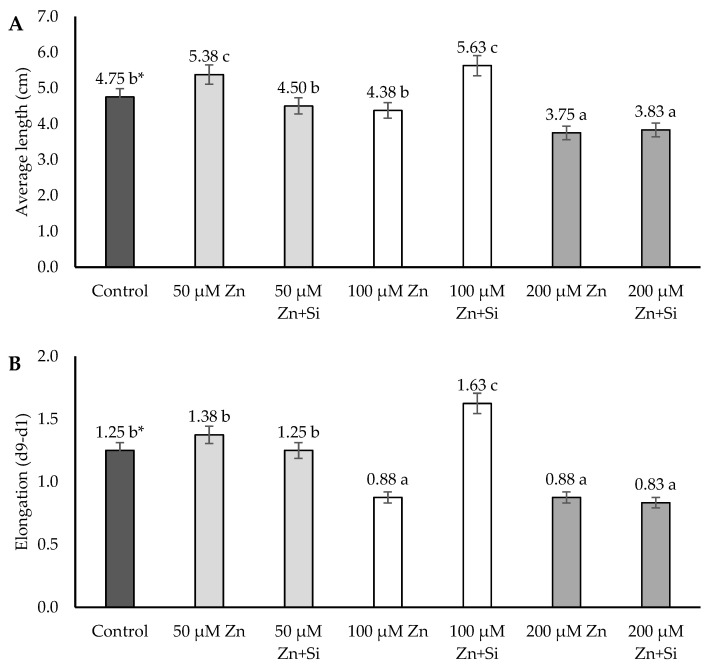
Effects of application of Zn and Si on the length (**A**) and growth (**B**) of the roots of barley; data are expressed as mean ± SD values. * Different letters indicate significant differences between the variants of the experiment (*p* ≤ 0.05).

In the case of root growth, a differential effect of Zn and Si was observed. Zinc ions lead to the inhibition as well as the stimulation of the growth parameters (Figure 2).

The elongation of the roots of barley plants was inhibited under high (200 μM Zn) and moderate (100 μM Zn) concentrations, whereas the average of the length was decreased only under a high Zn concentration. Interestingly, the stimulating effect of zinc was also observed at a low Zn concentration. This phenomenon was statistically confirmed only in the case of the average length parameter (Figure 2).

However, silicon application stimulated root growth only under moderate stress conditions induced by 100 μM Zn.

**Figure 3:**
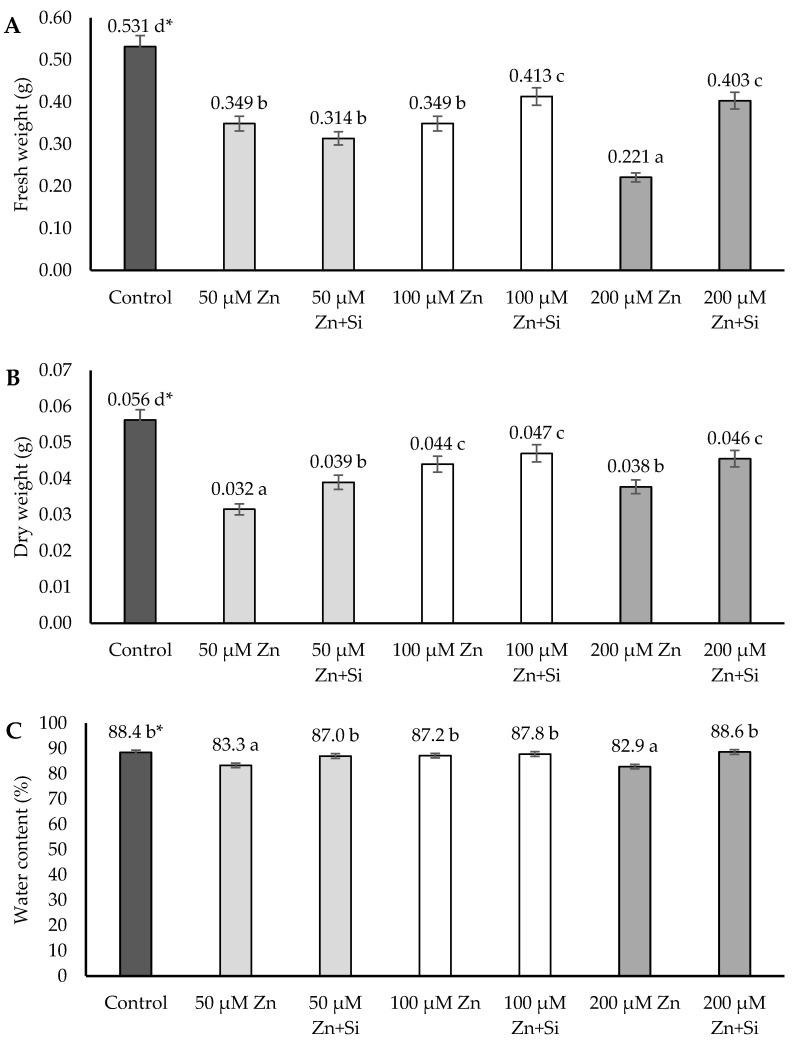
Effects of the application of Zn and Si on the fresh (**A**) and dry (**B**) weights, and the water content (**C**) of the barley’s above-ground parts; data are expressed as mean ± SD values. * Different letters indicate significant differences between the variants of the experiment (*p* ≤ 0.05).

The analysis of the fresh and dry weights of the barley plants and roots showed mainly inhibitory impacts of Zn, with some exceptions. The inhibition effect of Zn on the above-ground parts of plants was detected in the case of each concentration of Zn (except for the water content parameter for 100 μM Zn) (Figure 3).

The application of Si in most cases led to an increase in the analyzed parameters, which was especially visible in the case of high concentrations of Zn ions (Figure 3). The application of Si for plants under moderate stress conditions (100 μM) only stimulated the fresh weight of the plants, whereas under 50 μM Zn conditions, the dry weight as well as the water content were increased after Si application.

**Figure 4:**
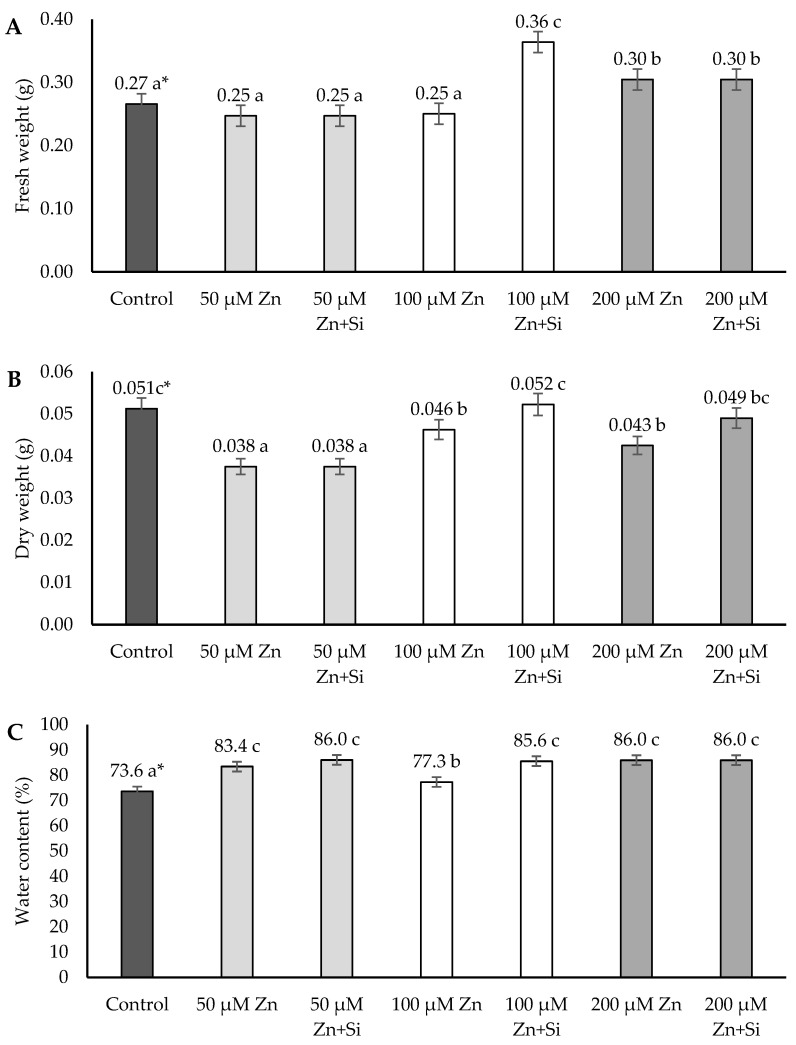
Effects of the application of Zn and Si on the fresh weight (**A**) and dry weight (**B**) and water content (**C**) of barley roots; data are expressed as mean ± SD values. * Different letters indicate significant differences between the variants of the experiment (*p* ≤ 0.05).

On the other hand, the root analysis shows the inhibition effect of Zn only in the case of the dry weight at all Zn concentrations (Figure 4).

The fresh weight and water content of the roots were not affected by Zn, and a stimulation effect was observed (Figure 4). The fresh weight increased in the case of a high concentration of Zn (200 μM Zn), whereas the water content grew in all Zn conditions (50 μM, 100 μM, and 200 μM Zn) (Figure 4). The application of silicon had a visibly positive effect on the roots of the barley plants under moderate stress conditions (100 μM Zn), whereas at low and high concentrations of Zn ions, silicon did not stimulate the analyzed parameters (except for the dry weight of the roots under 200 μM Zn conditions).

### 2.2. The Effects of Zn and Si Application on Plant Physiological Parameters

#### 2.2.1. Chlorophyll Content

**Figure 5:**
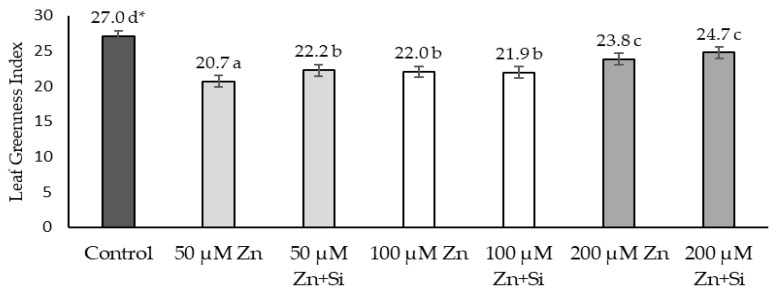
Effects of the application of Zn and Si on the relative chlorophyll content; data are expressed as mean ± SD values. * Different letters indicate significant differences between the variants of the experiment (*p* ≤ 0.05).

The reaction of Zn-induced stress conditions was also observed on a physiological level. Zn affected the relative chlorophyll content, leading to a decreased value in the leaf greenness index (SPAD). This result is significantly visible in the case of a low concentration of Zn (50 μM) as well as moderate and high Zn concentrations. However, only in a low-stress condition (50 μM Zn) was the stimulation effect of Si application confirmed. In the case of other Zn concentration conditions (100 and 200 μM), the stimulating impact of silicon was not statistically proved (Figure 5).

#### 2.2.2. Chlorophyll Fluorescence

**Figure 6:**
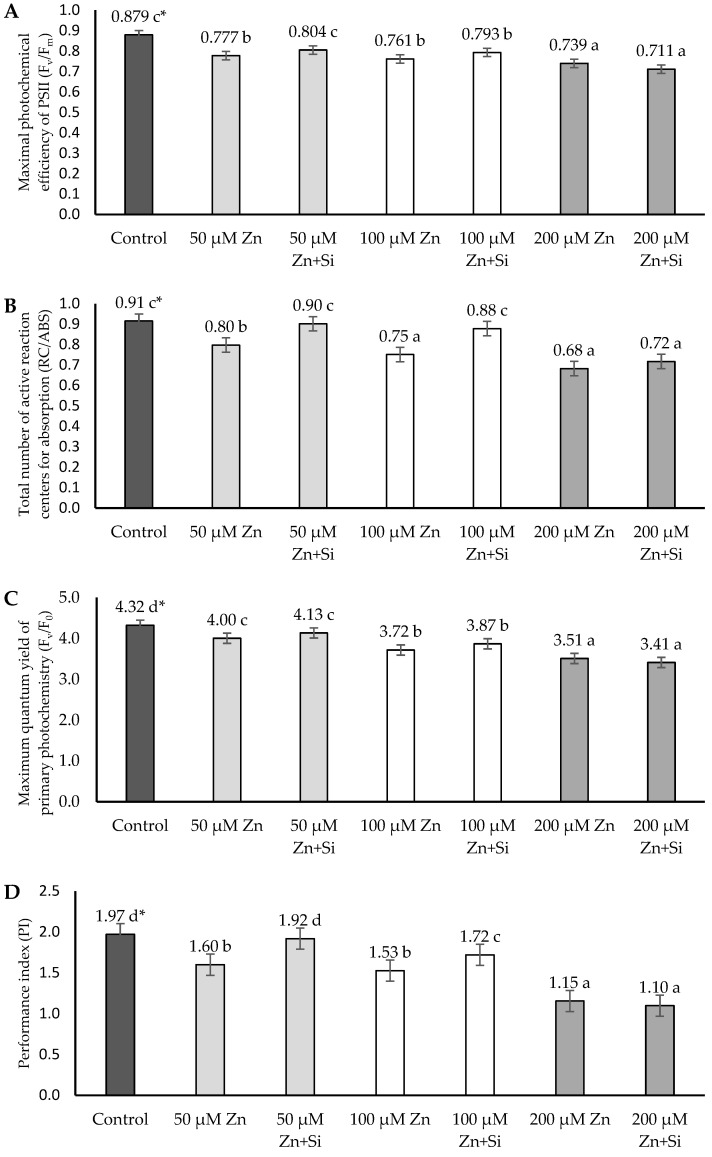
Effects of Zn and Si application on chlorophyll fluorescence parameters: maximum photochemical efficiency of PSII (F_v_/F_m_) (**A**), total number of active reaction centers for absorption (RC/ABS) (**B**), maximum quantum yield of primary photochemistry (F_v_/F_0_) (**C**), and performance index (PI) (**D**) in barley plants. Data are expressed as mean ± SD values. * Different letters indicate significant differences between the variants of the experiment (*p* ≤ 0.05).

The variance analysis showed a negative effect of different doses of Zn on the chlorophyll fluorescence parameters (F_v_/F_m_, RC/ABS, F_v_/F_0_, and PI) (Figure 6).

The negative effect of Zn was demonstrated by analyzing the F_v_/F_m_ parameter. Each applied dose of Zn caused a decrease in the F_v_/F_m_ parameter compared to the control. The highest (12.9%) was demonstrated after applying 200 µM Zn. Lower doses of Zn (50 and 100 µM) also affected the F_v_/F_m_ index, but the decrease compared to the control was lower (11.7 and 15.4%). The application of silicon to plants where 50 µM Zn was applied caused a significant increase in the F_v_/F_m_ index. A positive effect of Si was demonstrated compared to plants without the application of Si after applying 50 µM Zn (3.5%). Such dependencies were not observed in plants with a Zn dose of 100 µM and 200 µM (Figure 6A).

Compared to the control, the RC/ABS ratio also decreased. The largest and most significant decrease (25.3%) was observed in the treatment with the Zn dose of 200 µM. In treatments with a lower dose of Zn (50 and 100 µM), a significant decrease in the RC/ABS was also observed. However, it was lower and amounted to 12.1 and 17.6%, respectively. The analysis of variance showed a positive effect of silicon on the parameter of chlorophyll fluorescence. A significant increase was observed in the treatments in which barley plants were subjected to a lower Zn dose (50 and 100 µM): an increase of 12.5 and 17.3%, respectively. In the treatment with the highest Zn dose (200 µM), a positive effect of silicon was also observed, but it was not statistically significant (Figure 6B).

Compared to the control (without Zn and Si), each Zn dose caused a significant decrease in the F_v_/F_0_ parameter. The largest was shown after applying 200 µM Zn (a reduction of 18.8% compared to the control). The decrease was smaller at lower Zn doses (50 and 100 µM Zn) and amounted to 7.4% and 13.9%, respectively. The foliar application of silicon mitigated the negative effect of Zn. This was observed especially at lower Zn concentrations (50 and 100 µM), but this relationship was not statistically confirmed (Figure 6C).

The PI index also decreased under the influence of Zn-induced stress. The decrease was observed in each experimental object. The largest decrease according to the control was shown in the plants with 200 µM Zn: 44.1%. In plants with 50 and 100 µM Zn, the decrease was lower and amounted to 18.8 and 22.4%, respectively. A positive effect of silicon on the chlorophyll fluorescence index was demonstrated at lower Zn doses (50 and 100 µM). The increase in plants without Zn application was 16.7 and 12.4%, respectively. Such dependencies were not observed in the plants with a Zn dose of 200 µM (Figure 6D).

### 2.3. The Effects of Zn and Si Application on the Epigenetic Response of Plants

For the Methylation-Sensitive Amplification Polymorphism (MSAP) markers technique, five combinations of selective primers were used. As a result, 307–337 of the total clear bands were amplified from the plants analyzed for barley. The number of bands obtained varied among the differentially treated plants.

**Figure 7:**
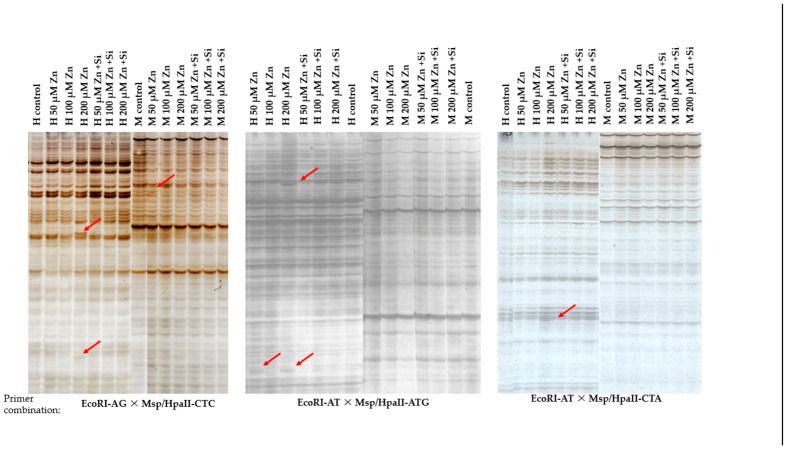
Sample of 3 electropherograms photo-presented DNA products of selective amplification with the selected primers used. Red arrows indicate polymorphic bands (products of selective amplifications). H and M signatures indicate separate bands for combination restriction enzymes EcoRI × HpaII and EcoRI × MspI, respectively, used in the restriction step of MSAP techniques.

A photo of an electropherogram showing the results of DNA methylation profiles is presented in Figure 7. As can be seen, a highly unlike band pattern of the amplification products was visible between the products of the enzyme pair EcoRI + HPaII (H-type bands) and the pair EcoRI + MspII (M-type bands) (Figure 7). Additionally, polymorphic products of selective amplification also occurred (Figure 7).

The overall frequency of DNA methylation was calculated (Table 1). The calculation is based on the number of bands (H-type and M-type) and their combination (1/0 or 0/1 for hemimethylation or symmetric ones) on the electropherograms.

The methylation level was different between analysis probes. The application of Zn as well as silicon-treated plants leads to a change in DNA methylation compared to that of the untreated control plants. Almost all of the analysis probes (Zn with or without silicon application) characterized decreasing total methylation. However, the lowest percentage was observed in plants that grew at a high Zn concentration (200 µM) (Table 1). It was observed both for silicon-sprayed plants and those without silicon (Table 1). The barley plants grew under low and moderate concentrations of Zn, also being characterized by a lower level of total methylation, but it was not as visible (Table 1).

An exception to these rules was the barley plants treated with a moderate concentration of Zn. In this group, the total methylation (%) was at the same level as that of the control plants (Table 1). The silicon application led to a modulation of the total methylation level compared to plants grown with the same Zn concentration but without Si application. The effect was dependent on the Zn concentration. The increase in the total DNA was distinguished in the case of low and high Zn concentrations (50 µM and 200 µM, respectively). As in the case of plants grown under moderate stress conditions (100 µM Zn), a decrease was observed (Table 1).

Other schemes and rules were observed for a particular type of methylation event (hemimethylation or symmetric ones). Silicon application leads to an increase in hemimethylation for the 50 µM and 100 µM Zn concentrations or a decrease for 200 µM Zn. In the case of symmetric methylation, the increasing and decreasing tendencies were the opposite. A group of plants grew under low and moderate Zn stress-induced conditions, being characterized by a higher frequency of symmetric methylation compared to silicon-sprayed plants. In the case of plants that grew under high Zn-induced stress conditions, the tendency was the opposite (Table 1).

## 3. Discussion

The polluting of heavy metals in soil and water has created a serious and growing environmental problem that has limited the growth and development of many crops. Plants lack an adaptive immune system, as in the case of humans and animals. Despite this, they can neutralize abiotic and biotic stresses by developing sophisticated strategies inside cells in response to stress [6]. Scientific research is, therefore, focused on finding solutions that allow plants to cope with unfavorable environments in a “better way”. One such solution is the use of silicon.

Investigations of the silicon effect on plants under stress conditions were carried out on various plant species such as cotton [28], maize [22,39,40], oat [23], wheat [41,42], and barley [21]. However, the research analyses were based mainly on the use of silicon in the form of solution application to soil. Such a solution also interferes with the natural environment by introducing additional components to the already-polluted soil (saturated with other elements, including phytotoxic heavy metals). Consequently, it carries the risk of physiological drought as a result of the high osmotic potential of the soil solution. Therefore, in the conducted studies, silicon was used in the form of a foliar spray to mitigate the effects of stress caused by Zn ions.

Many researchers have shown that Zn in high doses can cause various changes in plants, such as reduced growth and biomass, which is often observed under Zn toxicity conditions [1,7,8,13,14,15,16,43]. The limitation of plant growth is probably a consequence of lower Fe^2+^ and Fe^3+^ uptake and the inhibition of mitosis. Scientific studies report that excess Zn causes a significant reduction in the mitotic index. This reduction in mitotic activity may be due to the inhibition of DNA synthesis [7,12,17,44]. In addition, high levels of Zn damage the organization and lead to a decrease in nicotinamide (B vitamin), thus accelerating the breakdown of NAD+ and consequently reducing energy metabolism, which may be responsible for the decrease in the overall plant growth [12]. In the research carried out, barley plants grown under stress conditions mainly indicated a decrease in the morphological parameters of the above-ground parts of plants and less so for the roots, with some exceptions. The decrease was dependent on the Zn concentration as well as part of the plants (above ground or roots) and was visible especially in the case of a high dose of Zn (200 μM). On the other hand, the impact of Zn on roots was not so obvious. In the research presented, barley roots were affected by Zn stress conditions only at moderate and high doses of Zn (100 and 200 μM, respectively). The inhibition of root elongation as a result of Zn stress conditions was also observed by other researchers [8,12,18,19]. Besides a reduction in root growth [18], an affected root morphology [17,18], different length of the root hairs [18] and black and rotten root tips [12] were observed.

However, the analysis performed indicated that roots were also stimulated in reaction to Zn at a low dose (50 μM). The stimulation effect was visible, especially in the case of the average length (Figure 2) as well as the water content (Figure 4). The development of the root system is regulated by a complex and diverse signaling network, which, besides hormonal factors, includes reactive oxygen species (ROS) and nitrogen species (RNS). The delicate balance of the endogenous signal system can be affected by various environmental stimuli, such as an excess of essential heavy metals, like zinc (Zn) [45]. Zn at low concentrations can induce the morphological and physiological adaptation of the root system. Low Zn supplementation changes protein nitration patterns and stimulates root growth as a result [45]. This phenomenon was statistically proven in the performed research in the case of the average root length under 50 μM Zn stress-induced conditions (Figure 2).

Whereas a high Zn concentration increases nitrosative stress and nitration, it exerts toxic effects on plants by inhibiting root growth. The growth-inhibiting Zn treatment resulted in elevated protein tyrosine nitration due to an imbalance in ROS and RNS homeostasis. This nitro-oxidative stress was accompanied by serious changes in the cell wall composition and decrease in the cell proliferation and viability due to the high Zn uptake and disturbed microelement homeostasis in the root tips [45]. Silicon can counteract these negative effects of zinc stress-induced conditions.

The use of Si in stress conditions significantly improves plant responses to stress, which has been confirmed by many researchers [21,22,40,41,42]. Numerous studies have suggested the role of Si-mediated alleviation of abiotic stress through an enhancement in the antioxidant content and the upregulation of gene expression related to the suppression of ROS [46]. Si regulates the expression of genes encoding aquaporin (AQP), peroxidase (POX), phenylalanine ammonia-lyase (PAL), and pathogenesis-related protein (PR1) [47]. Additionally, Si increases the levels and activities of defense-related enzymes such as polyphenol oxidase (PPO), trypsin protease inhibitor, and peroxidase (POD), which are activated by Si in response to stress [48]. Furthermore, scientific studies have shown that the exogenous application of Si can improve the growth and development of plant crops experiencing stress from Zn toxicity by reducing Zn bioavailability [28]. Si minimizes metal toxicity by reducing ion absorption and translocation from the roots to the shoots [48]. Bhat et al. [24] reported that Si-mediated heavy metal stress tolerance includes chelation and the compartmentalization of metal ions, and toxic metals are co-precipitated along with the upregulation of antioxidants. This change causes gene regulation of metal transport-related genes, with overall structural alterations in plants.

The deposition of Si in the apoplast of the root cell wall leads to the blocked entry of heavy metals to the root cell wall and to other cells. Furthermore, the co-precipitation of Si and heavy metals shows a negative correlation with respect to the amount of heavy metals in different organs of plants due to reduced heavy metal uptake [24,48]. This was confirmed by Anwaar et al. [28], who indicated that the application of Si in cotton significantly inhibited the accumulation of Zn in different parts of the plants, i.e., roots, stems, and leaves, and thus promoted biomass [28]. The role of Si in mediating the alleviation of abiotic stress was also confirmed through nutrient homeostasis, modifications in phytohormones level like jasmonic acid [47], as well as higher leaf water content [46]. Furthermore Frazao et al. [49] showed that Si application causes a decline in the C level, which results in more N and P absorption, thereby changing the C/N/P stoichiometry in sugarcane, resulting in increased biomass production.

The above evidence confirms the results obtained in the performed experiment. Barley plants under stress conditions after Si treatment indicated an increase in morphological parameters, but it was dependent on the part of the plans (above ground or roots) and Zn dose. The stimulation effect of the above-ground barley plants was mainly visible in the case of an average length and elongation parameters or the fresh weight (in the cases of 100 µM and 200 µM Zn) as well as the dry weight and water content (in the cases of 50 µM and 200 µM Zn). In the case of the root morphological parameters analyzed, the stimulating effect of Si application was visible only in the case of moderate stress conditions (100 µM Zn).

The toxicity of Zn ions also affects processes related to photosynthesis, which has been confirmed by Abedi et al. [1], Islam et al. [13], and Kaya et al. [40]. This dependence was also observed in the presented research. Scientists [17,44] have reported that high doses of Zn lead to the loss of plasma membrane integrity and reduced biomembrane permeability, leading to leaf chlorosis and impaired photosynthesis. Ramakrishna and Rao [50] showed, in their study, a decrease in the content of photosynthetic pigments in sorghum, and this effect was dependent on the concentration of Zn. Similar conclusions were also presented by Kaya et al. [40]. Paunov et al. [51] showed a decrease in the chlorophyll content in durum wheat leaves after 7 days of treatment with 600 μM Zn. The presented research confirmed these reports (Figure 5).

To study the impact of many environmental stress factors on photosynthesis processes, a useful tool is the chlorophyll fluorescence technique (Chl). There are many fluorescence studies performed under abiotic stress conditions, such as increased soil salinity, drought, low or high temperature, or heavy metals in the soil [52,53,54,55,56,57]. The measurement of the photochemical process at the PSII level and the content of photosynthetic pigments provide information on the stress the plant is going through [58,59,60]. Studies on the influence of metals on the photosynthetic apparatus of plants indicate that the site particularly sensitive to the action of heavy metals is photosystem II (PS II). The influence of heavy metals, including Zn, is based on the inhibition of electron transport at the stage of absorption of an electron from a water molecule. Direct inactivation of the photosystem II reaction center under the influence of mercury, nickel, and chromium has also been demonstrated, as well as the influence of copper on many stages of the electron transport chain in the photosynthesis process [61,62]. An excess of Zn, besides changes in photosynthetic pigments, can cause photosynthesis disorders at various structural and functional levels of the cell. This includes changes in light capture, the ultrastructure of the thylakoid and photosynthetic electron transport, stomatal conductance and the availability of CO_2_, as well as enzyme activity in the Calvin cycle [63]. Paunov et al. [51] indicated the migration of energy from antenna complexes to chlorophyll reaction centers, which leads to increased chlorophyll fluorescence emission in the dark-adapted parts of durum wheat leaves after 7 days of treatment with 50 μM Cd and 600 μM Zn.

In rice, heavy metals reduce the F_v_/F_m_ and values of F_v_/F_0_ [64] as well as lead to lower PSII efficiency [14]. Similarly, Vassilev et al. [65] showed a negative effect of Zn on the photosynthetic apparatus of beans.

Our studies conducted on barley also showed a negative effect of the applied Zn doses. Compared to the control, a decrease in the chlorophyll fluorescence parameters (F_v_/F_m_, F_v_/F_0_, RC/ABS, and PI) was observed in each treatment (50, 100, and 200 μM Zn). The largest decrease was observed at 200 μM Zn. The negative effects of heavy metals on photosynthesis can be reduced by the application of silicon. According to Song et al. [4] the positive role of Si in increasing the tolerance to Zn-induced stress is due to increased cell photosynthesis and antioxidant capacity.

Si mediated the chelation and compartmentalization of metal ions, which led to the co-precipitation of toxic metals along with the upregulation of antioxidants. This change causes the regulation of metal transport-related genes, with overall structural alterations in plants [24]. Scientific studies suggested that Si is capable enough to maintain the uneven swelling and disintegrated and missing thylakoid membranes caused during stress [46]. Improvements in chlorophyll fluorescence parameters induced by Si addition were reported in cucumber [64], rice [66] (heavy metals), tomato (salt stress) [67], and sorghum (drought stress) [68]. In our study, a positive effect of Si was observed in barley plants exposed to stress caused by Zn. A statistically significant increase was proved in the case of lower doses of Zn (50 µM). A positive effect of Si was also observed at the highest Zn dose (200 µM Zn), but it was not statistically confirmed. An improvement in the chlorophyl pigment after Si application has also been reported by many researchers [46,47,66].

Plant response to Zn-induced stress depends not only on the concentration of the stressor but also on the species of plants [69,70] or the variety [71]. This difference may result from the adaptive capacity/response of plants to stress factors. The basis of this “ability” is epigenetic changes. Numerous scientific studies show that the change in gene expression that occurs under the influence of stressful conditions is the result of epigenetic changes [33,72]. Plants change their epigenetic profile to adapt to or overcome stress conditions. A change in the methylation level may generate acquired resistance to stress conditions. This acquired resistance, named “epigenetic memory”, may be transmitted to the offspring as a result of generative reproduction [33,73]. DNA methylation analyses of barley in response to stress conditions were performed in the case of different stress conditions. Stadnik et al. [21] indicated a different pattern of DNA methylation of plants subjected to salinity stress compared to the control. The effect of methylation changes was NaCl dose dependent. The studies carried out showed a change in the level of methylation in both plants subjected to Zn-induced stress and plants that were also sprayed with silicon. Differences were also visible between the total methylation frequency and hemi- or symmetric methylation. Similar observations were detected by Stadnik et al. [21]. According to Dorts et al. [74], DNA methylation is one of the first and most studied regulatory mechanisms discovered in epigenetics and is considered a relatively stable, heritable, and transgenerational mark, involving a series of biological processes such as temporal and spatial gene expressions, transposable element activity, and genomic imprinting [34,75]. The difference between plants sprayed with Si and those that were not treated with Si indicates that silicon affects the epigenome of barley plants, thereby modifying the response of plants to stress factors at the molecular level. This modification may be the basis for plants to acquire resistance to stress conditions [72], which will be passed on to offspring cells [33]. The creation of “epigenetic memory” in response to stress will allow the next generation of plants to “cope”/adapt to stress conditions in a better way.

## 4. Materials and Methods

The research was carried out on barley plants (*Hordeum vulgare* L.) grown under hydroponic conditions. The analysis consists of three parts: morphological, physiological, and epigenetic.

### 4.1. Plant Material

The seedlings of the barley used for the research were obtained via germination of the selected spring barley seeds. The seeds were sterilized by treating them in the following solutions: 1–70% ethyl alcohol (20 s); 2–20% sodium hypochlorite solution (10 min); and 3 times distilled water (5–10 min.). Afterwards, they were germinated on Petri dishes containing lignin in high-humidity conditions (about 90%) and at a temperature of 18–22 °C. Subsequently, 5-day seedlings (containing 3 leaves) were used for hydroponic cultivation.

#### 4.1.1. Hydroponic Cultivation

The seedlings were grown in Erlenmeyer flasks (130 mL of total volume) under hydroponic conditions. Hoagland liquid medium (with some modifications) was used for hydroponic cultivation [76]. The pH of the nutrient solution was adjusted to 6.2 using HCl. Zn stress-induced conditions were generated by adding to the Hoagland solution ZnS0_4_ × 7H_2_O at concentrations of 50 µM, 100 µM, and 200 µM Zn, at 100 mL each of the liquid medium combination solution (control Hoagland medium; Hoagland medium + 50 μM Zn; Hoagland medium + 100 μM Zn; Hoagland medium + 200 μM Zn) was poured into Erlenmeyer flasks (8 replicates for each type of medium). The suitable medium solutions were refilled at two-day intervals. Three barley seedlings were placed in each of the Erlenmeyer flasks, with the roots dipped in the liquid medium. Sterile cotton wool was used as a platform for the seedlings, enabling the immersion of the roots into the liquid medium as well as for air circulation. Barley seedlings were exposed to different Zn stress conditions for two days and treated with silicon afterward. For this purpose, the plants were divided into 2 groups: seedlings growing under Zn-induced stress, treated with Si, and seedlings growing under Zn-induced stress, not treated with Si.

Finally, each group consisted of 4 Erlenmeyer flasks with 3 seedlings for four types of medium combinations. In total, 12 seedlings for any analyzed medium combination in both groups (Si treated and untreated) were used. The additional control group consisted of seedlings (12) growing under no-stress conditions, on the Hoagland solution. A 0.1% preparation of silicon (Optysil ^®^, Intermag Sp. z o.o., Olkusz, Poland) containing SiO_2_ (200 g ‧ L^−1^) was used to hand spray the barley seedlings on the first day of the experiments. Spraying was conducted with a laboratory hand sprayer with a flow control of a dosing volume of 1.2 mL ± 0.1 during one press (outlet diameter of 0.6 mm). This was applied via a uniform spraying procedure.

#### 4.1.2. Growing Conditions

All the plants analyzed were grown in phytotron growth chambers under the following conditions: 22–24 °C temperature; 16/8 h (light/darkness) photoperiod; and 12 μmol∙m^−2^∙s^−1^ PPFD.

The measurement analysis was started one day after spraying Si and was finished on the 9th day. During the experiment, the length and relative chlorophyll content were measured on the 1st, 3rd, 5th, 7th, and 9th days, whereas the fresh weight and fluorescence parameters were measured on the last day (9th) of the experiment. Additionally, at the end of the experiment, the plant material was collected for further analysis of the dry weight, water content, and DNA methylation.

### 4.2. Morphological Analysis

Morphological analysis included the length measurement (leaves and roots), elongation, dry and fresh weight measurements, as well as the water content (%). To analyze the length of the above-ground plants, only leaves with a minimum 3 cm value were included. The shoot measurements were made at the beginning of the experience (d1) and on the seventh day. The fresh weight (FM) and dry weight (DW) were determined by weighing the above-ground parts and roots collected at the end of the experience (d9). The dry weight was determined after drying the plant material at 65 ° C for 72 h, according to Huang et al. [77]. To evaluate the water content (%), the formula followed that of Huang et al. [77].

### 4.3. Physiological Analysis

Physiological analyses were based on the measurement of the relative content of chlorophyll, as well as the fluorescence. The relative chlorophyll content was recorded using a chlorophyll meter (SPAD-502 plus, Konica Minolta, Osaka, Japan). Measurements were used for 4 representative plants from each type of medium combination, treated and untreated with Si. The measurement was performed in 3 replicates for each of the analyzed plants.

Chlorophyll fluorescence transients were measured with a plant efficiency analyzer (Handy PEA, Hansatech Ltd., Norfolk, UK). Measurements were made on the upper surfaces and the middle portion of the first leaves formed, following a dark adaptation period of 20 min, using leaf clips. The fluorescence signal was collected under actinic red light, with a light source peak wavelength of 627 nm, and transmitted for 1 s at the maximum available intensity of 3500 μmol (photon) of photosynthetically active radiation (PAR) m^2^ s^−1^. Analyses were performed in 2 replicates for the leaves of 4 representative plants from each types of medium combination, treated and untreated with Si. The following parameters were determined: the maximal photochemical efficiency of PSII (F_v_/F_m_), the total number of active reaction centers for absorption (RC/ABS), the maximum quantum yield of the primary photochemistry (F_v_/F_0_), and the performance index (PI).

### 4.4. Epigenetic Analysis

To detect the impact of silicon on plants treated with Zn at different concentrations, Methylation-Sensitive Amplification Polymorphism (MSAP) markers were used. For epigenetic analysis, DNA isolation from the bulk of leaves was performed using each of the probes. The isolation was conducted according to the Doyle and Doyle protocol [78].

The purpose of isolation was to obtain high-quality DNA at a concentration 50 ng/µL (absorbance at λ260/280 in the range of 1.8–2.2). For the MSAP technique, based on the Peraza-Echeverria et al. [79] and Xiong et al. [80] protocol, 4 separate reactions are included: (1) restriction, (2) ligation of adapters, (3) pre-amplification, and (4) selective amplification. Subsequently, primers (or adapters) according to Peraza-Echeverria et al. [79] were used. Five pairs of selective primer combinations were applied for MSAP analysis. The sequence of selective primers is presented in Table 2. The reaction was carried out under the following conditions and times: restriction for 2 h at 65 °C; ligation at 37 °C for 6 h. The PCR conditions (pre-amplification and selective amplification were as follows: 94 °C for 1 min, 65 °C for 1 min, and 72 °C for 1 min for 35 cycles and final extension at 72 °C for 7 min for 1 cycle. For selective amplification, the annealing of the starters was at 65 °C with touch down (in each successive cycle, the temperature was reduced by 0.7 °C). Each of the reactions was performed in a thermocycler (Biometra, Göttingen, Germany). The PCR-selective products obtained were subjected to electrophoresis and then separated on a 6% denaturing polyacrylamide gel. The gels were stained with silver nitrate [81] and further scanned for data recording. The MSAP analysis was performed with two or more repetitions, depending on the quality of the bands obtained.

### 4.5. Methylation Analysis

The enzymes HpaII and MspI (Thermo Scientific, Waltham, MA, USA), used for the MSAP analysis, are isoschizomers that recognized the same sequence of DNA and cut the dependency of the methylation occurring.

The methylation analysis was performed according to the information presented by Xiong et al. [80]. The presence of bands in the gel of the reaction EcoRI + MspI (M) and their absence from the reaction EcoRI + HpaII (H) simultaneously indicated DNA methylation. In this situation, internal cytosine from the 5′CCGG 3′ sequence was methylated (5′CmCGG 3′). This is considered as “symmetric or fully methylation”. In contrast, the absence of bands in the gel from the reaction EcoRI + MspI (M) and their presence in the reaction EcoRI + HpaII (H) simultaneously indicated DNA methylation, also. However, in this situation, the external cytosine of one DNA strand was methylated (5′mCCGG 3′). This occurrence was described as the “hemimethylated state”. The frequency of methylation was calculated following Xiangqiana et al. [82], as follows:

$$Metylation\;\left(\%\right)=\frac{number\;of\;methylated\;bands}{total\;number\;of\;bands}\times 100$$

### 4.6. Statistical Analysis

Statistical analysis was performed using the TIBCO Statistica 13.3.0 package (TIBCO Software Inc., Palo Alto, CA, USA). The Shapiro–Wilk test was performed to detect deviations from the normal distribution at *p* = 0.05. The homogeneity of variance was checked. Then, an analysis of variance (AVOVA) was performed. To determine and verify the relationships, Fisher’s LSD post hoc test was performed with a significance level of *p* ≤ 0.05.

## 5. Conclusions

The growing threat of polluted soil and the environment brings the necessity for a solution that will allow plants to cope with environmental stress conditions. It is necessary to find a solution that can eliminate the negative effects of stress caused by polluted soils, including heavy metals, without adding new nutrients into the saturated soil solution. One of these resolutions is the application of silicon as a foliar spray. The spraying of silicon on barley plants helped mitigate the inhibitory effect of Zn-induced stress. This was observed in the morphological as well as physiological levels, whereas the mitigating effect of Si was dependent on the Zn dose as well as the part of plants (above ground or roots). The epigenetic analysis confirmed different DNA methylation levels between plants that grew under Zn stress, either Si treated or untreated. It could be assumed that the modification of the plant response after Si treatment is a consequence of epigenetic rearrangements. But to prove this hypothesis, an investigation of the expressions of specific gene needs to be performed. However, epigenetic rearrangements can lead to the creation of “epigenetic memory”. Plants with this “epigenetic memory” will cope with stress better the next time. Nonetheless, to confirm this phenomenon, further analysis on other plant species, as well as offspring, obtained from stress-treated plants is needed in the future. Additionally, future studies are needed to thoroughly investigate the mechanisms of Si-mediated heavy metal tolerance or for monitoring Si and Zn incorporation into tissues and cells, as well as the analysis of particular gene expressions.

## Figures and Tables

**Table 1:** Results of MSAP analysis.

Parameters Analyzed:	Control	50 µM Zn	50 µM Zn + Si	100 µM Zn	100 µM Zn + Si	200 µM Zn	200 µM Zn + Si
Total band number	307	330	328	328	329	337	329
Number of symmetric methylation bands	56	54	53	54	52	52	53
Symmetric methylation (%)	18.2	16.4	16.2	16.5	15.8	15.4	16.1
Number of hemimethylation bands	75	84	85	86	87	85	82
Hemimethylation bands (%)	24.4	25.5	25.9	26.2	26.4	25.2	24.9
% total methylation	42.7	41.8	42.1	42.7	42.2	40.7	41

**Table 2:** Sequences of selective primers used for selective amplification.

Primer Name	Sequence
EcoRI-ACT	5′GACTGCGTACCAATTCACT3′
EcoRI-AC	5′GACTGCGTACCAATTCAC3′
EcoRI-AT	5′GACTGCGTACCAATTCAT3′
MspI/HpaII-ATG	5′GATGAGTCCTGAGTCGGATG3′
MspI/HpaII-CTA	5′GATGAGTCCTGAGTCGGCTA3′
MspI/HpaII -CTC	5′GATGAGTCCTGAGTCGGCTC3′
MspI/HpaII- CT	5′GATGAGTCCTGAGTCGGCT3′

## Data Availability

The data presented in this study are available from the corresponding author upon reasonable request.
